# Effects and Usefulness of Inspiratory Muscle Training Load in Patients with Advanced Lung Cancer with Dyspnea

**DOI:** 10.3390/jcm12103396

**Published:** 2023-05-10

**Authors:** Yasunari Sakai, Takayoshi Yamaga, Shuhei Yamamoto, Keiji Matsumori, Takashi Ichiyama, Masayuki Hanaoka, Shota Ikegami, Hiroshi Horiuchi

**Affiliations:** 1 Department of Rehabilitation, Shinshu University Hospital, Matsumoto 390-8621, Japan; shuhei.0703@gmail.com (S.Y.); keiwbc1@shinshu-u.ac.jp (K.M.); horiuchih@shinshu-u.ac.jp (H.H.); 2 Department of Occupational Therapy, Faculty of Medical Science, Nagoya Women’s University, Nagoya 467-8610, Japan; serisaito623@gmail.com; 3 Department of Respiratory Center, Shinshu University Hospital, Matsumoto 390-8621, Japan; ichiyama@shinshu-u.ac.jp (T.I.); masayuki@shinshu-u.ac.jp (M.H.)

**Keywords:** advanced lung cancer, inspiratory muscle training, maximal inspiratory pressure, dyspnea

## Abstract

Background: Patients with advanced lung cancer tend to experience dyspnea. Pulmonary rehabilitation has been reported as a method for relieving dyspnea. However, exercise therapy imposes a high burden on patients, and it is difficult to sustain in many cases. Inspiratory muscle training (IMT) imposes a relatively low burden on patients with advanced lung cancer; however, its benefits have not been demonstrated. Methods: We retrospectively analyzed 71 patients who were hospitalized for medical treatment. The participants were divided into an exercise therapy group and an IMT load + exercise therapy group. Changes in maximal inspiratory pressure (MIP) and dyspnea were examined using a two-way repeated measures analysis of variance. Results: MIP variations significantly increase in the IMT load group, with significant differences between baseline and week 1, between week 1 and week 2, and between baseline and week 2. The analysis also showed that the variations in dyspnea decreased in the IMT load + exercise therapy group with significant differences between baseline and week 1 and between baseline and week 2. Conclusions: The results show that IMT is useful and has a high persistence rate in patients with advanced lung cancer who present dyspnea and cannot perform high-intensity exercise therapy.

## 1. Introduction

Common symptoms of lung cancer include dyspnea, cough, weight loss, and chest pain [1]. Research shows that approximately three-quarters of patients with lung cancer experience dyspnea, with approximately 90% developing dyspnea the month before their death [2]. In particular, patients with advanced lung cancer, who inevitably require frequent or prolonged hospitalization, are susceptible to worsening dyspnea at rest and on exertion. Moreover, the vicious cycle of symptoms such as chronic dyspnea and fatigue associated with reduced activity is predicted to reduce exercise tolerance and quality of life (QOL). Regardless of the type of lung cancer treatment, cancer infiltration into the lung and surrounding tissues interrupts breathing and can cause dyspnea at rest and on exertion. Regardless of the status of the lung parenchyma, patients with advanced cancer may experience dyspnea caused by malaise and respiratory muscle fatigue [3].

The American Society of Clinical Oncology (ASCO) guidelines recommend a hierarchical approach to dyspnea, with reversible causes first, followed by non-pharmacological and pharmacological interventions as the last addition to the treatment plan and from those with fewer side effects and higher efficacy than those with a higher potential for side effects [4]. Pulmonary rehabilitation provided to individuals with chronic respiratory diseases (i.e., interstitial lung disease, bronchiectasis, cystic fibrosis, asthma, pulmonary hypertension, lung cancer, lung volume reduction surgery, and lung transplantation) has demonstrated improvements in symptoms, exercise tolerance, and QOL [5]. The National Institute for Health and Clinical Excellence has recommended non-pharmacological treatment as the standard of care for dyspnea in patients with lung cancer [2]. In particular, pulmonary rehabilitation, which includes patient education, exercise therapy, respiratory muscle training, and nutritional therapy, has been found to be useful in improving dyspnea in patients with lung cancer [6,7,8]. Although limited in number, interventions have focused on the usefulness of exercise therapy as an element of pulmonary rehabilitation in patients with advanced lung cancer with dyspnea [9]. In addition, exercise therapy has been widely reported as an element of pulmonary rehabilitation in patients with respiratory diseases, mainly chronic obstructive pulmonary disease (COPD), and a high-intensity exercise load has been recommended [10,11]. However, it is very difficult for patients with advanced lung cancer to have difficulty maintaining active exercise therapy and activities due to dyspnea [12], which explains the low persistence rate of high-intensity exercise therapy. On the other contrary, inspiratory muscle training (IMT) has been used as a non-pharmacological intervention for respiratory symptoms since the 1980s [13]. IMT, also known as respiratory or ventilatory muscle training, aims to improve inspiratory muscle strength and endurance through a series of breathing exercises. Improving inspiratory muscle strength and endurance is a management strategy that may help relieve symptoms of dyspnea, thereby increasing the level of activity and improving the QOL of patients with respiratory problems. Moreover, the benefits of IMT for dyspnea have been studied in patients with COPD, heart failure, and restrictive thoracic disease [14,15,16]. IMT can strengthen the inspiratory muscles, and stronger inspiratory muscles require less effort during a given task; thus, dyspnea is reduced [17]. Controlled breathing can also improve dyspnea [18], and IMT is one such method.

In 2015, a study reported the efficacy of IMT loading for patients with advanced lung cancer without indication for surgery, and it was shown to be effective in improving dyspnea and QOL [19]. However, very few reports have validated IMT loading for patients with advanced lung cancer without indication for surgery. Therefore, this study aimed to investigate the effects and benefits of IMT loading on patients with advanced lung cancer and dyspnea.

## 2. Materials and Methods

### 2.1. Study Population

**Figure 1:**
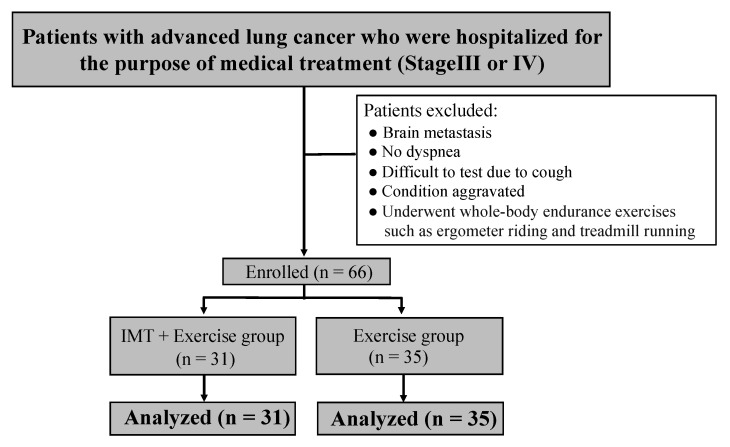
Flow chart of study participation. A total of 71 subjects were targeted for investigation during the study period. After the exclusion criteria were applied, 66 subjects were included in the analysis.

This retrospective cohort study included patients with advanced stage III–IV lung cancer who were hospitalized for medical treatment and underwent rehabilitation between April 2015 and March 2019 (Figure 1). The exclusion criteria were as follows: patients who participated in whole-body endurance exercises such as ergometer riding and treadmill running, which are considered effective in improving dyspnea; patients without dyspnea at rest or on exertion (modified Borg scale [mBS] = 0); patients in whom test reliability could not be ensured because of cough; patients with brain metastases. Patients were stratified according to their rehabilitation status upon admission into two groups: those who received only exercise therapy (control group) and those who were provided IMT + exercise therapy group (intervention group). 

### 2.2. Characteristics of the Patients

Patient age, sex, body mass index, cancer type and stage, performance status, Barthel index, mBS rating as the degree of dyspnea at rest and on exertion (usual activities of daily living situations in hospital wards), type of chemotherapy, line of therapy, use of oxygen therapy, spirometry, and maximal inspiratory pressure (MIP) were retrospectively analyzed. The percentage of vital capacity (%VC) forced expiratory volume in 1 s and forced vital capacity (FVC) were measured using an Autospiro AS-407 spirometer (Minato Medical Science, Osaka, Japan) based on a previous study [20]. Relevant data, including daily rehabilitation evaluations, were obtained from medical charts. In addition, relevant data from medical charts were obtained by medical staff who were unrelated to this study and were blinded. Due to follow-up loss, 66 participants were included in the analysis.

### 2.3. Outcome Measures

The mBS rating and MIP were indicators of dyspnea. Therefore, the mBS rating and MIP were collected at three time points: baseline, 1 week later, and 2 weeks later. The mBS is a subjective rating scale that measures the degree or intensity of dyspnea at rest and on exertion. Dyspnea on exertion was assessed when it was felt most strongly during the day’s activities. The mBS is a 12-point scale ranging from 0 (none) to 10 (maximum) plus 0.5 and is also called the Clinical Rating Scale for Dyspnea (CR-10). Its score ranges from 0 to 10 (maximum). The mBS scale was adopted because it is considered appropriate for assessing relative changes in dyspnea.

MIP was measured as previously described [20] using the POWERbreathe Medic Plus KH2 (Entry Japan, Tokyo, Japan). Maximum inspiration was determined from the residual air volume in the sitting position, and the pressure was maintained for 3 s. The measurements were performed three times, and the maximum values were used for the analysis [21].

### 2.4. Rehabilitation Program

All rehabilitative strategies were carried out in daily sessions during their hospital stay (Monday through Friday). Daily sessions lasted 20–40 min for each rehabilitative intervention. Owing to the characteristics of this university hospital, the hospital stay is approximately 2 weeks, so the rehabilitation program is also set at 2 weeks. A pressure threshold device, commercially available as POWERbreathe Medic Plus KH2 (Entry Japan, Tokyo, Japan), was used to provide IMT. It includes a mouthpiece and a calibrated spring-loaded valve that controls a constant inspiratory pressure training load that is maintained unless the patient drastically changes his breathing pattern. Exercise therapy did not include whole-body endurance exercises, such as riding an ergometer or treadmill running, because this study was designed for patients with severe respiratory symptoms. Exercise therapy consisted mainly of activities of daily living training, including respiratory muscle stretching, upper and lower limb resistance training, basic movements, and walking, under the guidance of physical therapists. The initial training workload was chosen to start at 0 and gradually increase until the patient-rated dyspnea and/or leg fatigue as 4 or 5 on an mBS. All patients received the same exercise therapy.

IMT is believed to improve tolerance not only in the early phase of training but also in patient satisfaction and adherence by not inducing significant dyspnea. The IMT load + exercise therapy group performed IMT as previously described [19,21,22] up to 30 breaths twice a day (5 days a week) at a load of 30–40% of the MIP, with nose clips attached and under the appropriate guidance of physiotherapists. We have adjusted the MIP according to the new MIP values measured each week. However, initially, sessions had to be shorter than 30 breaths if patients had difficulty completing the entire session because of fatigue or breathlessness. The MIP was then measured using the POWERbreathe Medic Plus KH2, and the load was adjusted each week to the optimal load.

### 2.5. Statistical Analysis

The clinical backgrounds of the exercise therapy and the IMT load + exercise therapy groups were compared using the *χ*^2^ test and Student’s *t*-test. Variations in MIP and MBS ratings were examined using a two-way repeated-measures analysis of variance (ANOVA) to investigate the effects of IMT. Mauchly’s test of sphericity was used to test the assumption of sphericity; when it yielded statistically significant results, the Greenhouse–Geisser *ε* correction was used to adjust violations of sphericity. In addition, Bonferroni’s method was used for multiple comparisons. Statistical analysis was performed using EZR software (EZR Version 1.60., The R Foundation for Statistical Computing, Vienna, Austria) [23]. Descriptive data are expressed as the mean ± standard deviation or standard error values. The significance level was set at 5%.

## 3. Results 

### 3.1. Adherence

**Figure 2:**
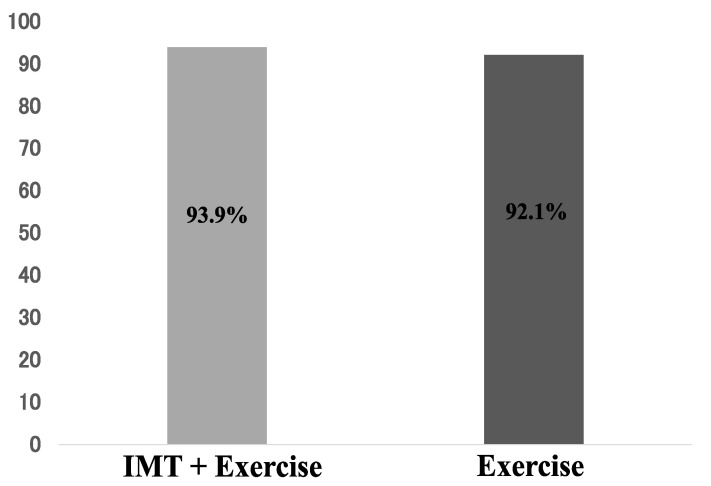
Treatment continuation rate of each group.

Three patients from the exercise therapy group and two patients from the IMT load + exercise therapy group dropped out of this study because of a deteriorated general condition caused by the primary disease. None of the patients withdrew from this study due to the IMT load. The difference in the persistence rates between the two groups, with 92.1% in the exercise therapy group and 93.9% in the IMT load + exercise therapy group, was not statistically significant (Figure 2). No difference in adherence was observed between the two groups.

### 3.2. Participant Characteristics

Table 1 presents the baseline clinical characteristics of the participants at baseline. The %VC, FVC, and MIP in the exercise therapy group were significantly higher than those in the IMT load + exercise therapy group. No significant differences were found for the other items measured.

### 3.3. Two-Way Repeated-Measures ANOVA 

**Figure 3:**
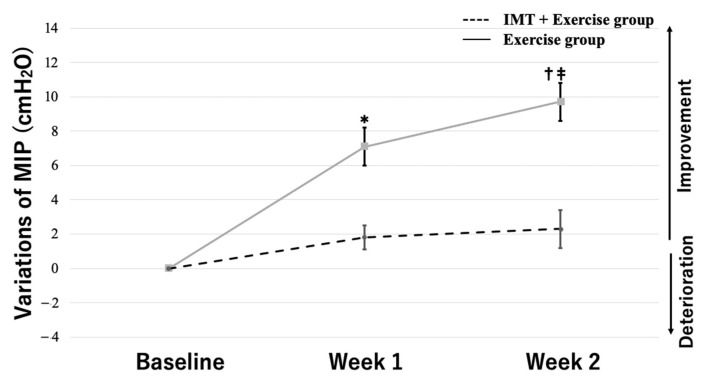
Effects of IMT on MIP. Data are mean ± SE. * *p* < 0.01 versus the combined IMT group at baseline. ^†^ *p* < 0.01 versus the combined IMT group in week 1. ^‡^ *p* < 0.01 versus the combined IMT group before intervention. T × G indicates the time × group interaction.

**Figure 4:**
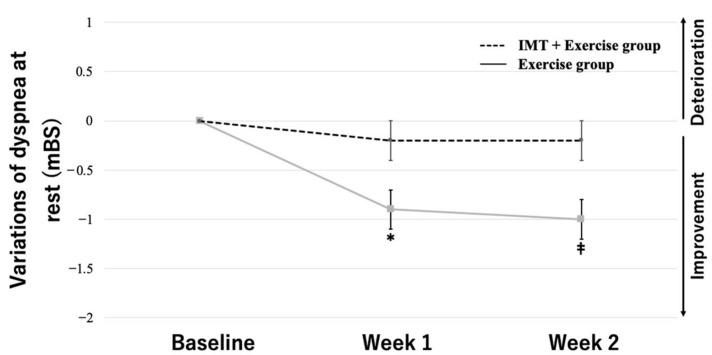
Effects of IMT on dyspnea at rest. Data are mean ± SE. * *p* < 0.01 versus the combined IMT group at baseline. ^‡^ *p* < 0.01 versus the combined IMT group before intervention. T × G indicates the time × group interaction.

**Figure 5:**
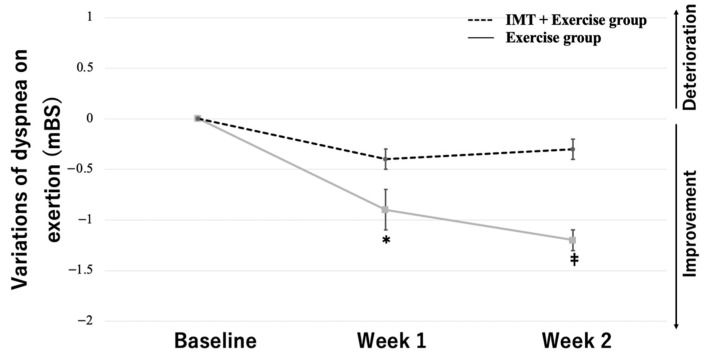
Effects of IMT on dyspnea on exertion. Data are mean ± SE. * *p* < 0.01 versus the combined IMT group at baseline. ^‡^ *p* < 0.01 versus the combined IMT group before intervention. T × G indicates the time × group interaction.

Based on the results of the two-way ANOVA with group and training period as the two factors, MIP variations showed a significant interaction between group and period (*F* = 17.14, *p* < 0.01). The multiple comparisons test showed that the MIP variations in the IMT load + exercise therapy group increased, with significant differences between baseline and week 1 (33.2 [±7.2] vs. 40.3 [±8.4]), between weeks 1 and 2 (40.3 [±8.4] vs. 42.2 [±8.4]), and between baseline and week 2 (33.2 [±7.2] vs. 42.2 [±8.4]) (*p* < 0.01 for all). In contrast, the MIP variations in the exercise therapy group did not show significant changes between baseline and week 1 (39.9 [±11.2] vs. 41.7 [±9.4]), between weeks 1 and 2 (41.7 [±9.4] vs. 42.2 [±8.9]), and between baseline and week 2 (39.9 [±11.2] vs. 42.2 [±8.9]) (Figure 3). The variations in dyspnea at rest and on exertion showed a significant interaction between group and period (*F* = 8.76, *p* < 0.01, at rest; *F* = 13.40, *p* < 0.01, on exertion). The multiple comparisons test showed that the variations in dyspnea at rest in the IMT load + exercise therapy group decreased, with significant differences between baseline and week 1 (2.3 [±0.9] vs. 1.4 [±0.9]) and between baseline and week 2 (2.3 [±0.9] vs. 1.3 [±1.0]) (*p* < 0.01 for both). In contrast, the variations in dyspnea at rest in the exercise therapy group did not show significant changes between baseline and week 1 (2.0 [±1.0] vs. 1.9 [±1.2]), between weeks 1 and 2 (1.9 [±1.2] vs. 1.8 [±1.9]), or between baseline and week 2 (2.0 [±1.0] vs. 1.8 [±1.9]) (Figure 4). Moreover, the variations in dyspnea on exertion in the IMT load + exercise therapy group decreased, with significant differences between baseline and week 1 (3.8 [±0.8] vs. 2.9 [±1.0]) and between baseline and week 2 (3.8 [±0.8] vs. 2.6 [±0.9]) (*p* < 0.01 for both). In contrast, the variations in dyspnea on exertion in the exercise therapy group did not show significant changes between baseline and week 1 (3.9 [±1.0] vs. 3.6 [±1.3]), between weeks 1 and 2 (3.6 [±1.3] vs. 3.6 [±1.3]), or between baseline and week 2 (3.9 [±1.0] vs. 3.6 [±1.3]) (Figure 5).

## 4. Discussion

Notably, a few randomized controlled trials (RCTs) on dyspnea in patients with advanced lung cancer are adequately powered. Currently, no previous studies have limited their inclusion criteria to patients with advanced lung cancer or focused on short-term effects. In this present study, the results showed that IMT load significantly improved MIP and dyspnea at rest and on exertion in the participants. In addition, IMT is extremely beneficial and has a high persistence rate in patients with advanced lung cancer. Notably, IMT load improved MIP and dyspnea relatively early. Therefore, this study is extremely rare, as the results suggest that IMT loading may be useful as a non-pharmacological treatment for dyspnea in patients with advanced lung cancer who have received only medical treatment alone. We assume that this study referred to a situation in which the load intensity and time of IMT use were somewhat less than those in RCTs of patients with thoracic malignancies [19] because we wanted to keep patient adherence as high as possible. However, the effectiveness of IMT in this situation not only increases its usefulness but also may have influenced the high adherence of patients, as revealed in the results of this study. Traditionally, IMT has been considered extremely useful in patients with reduced respiratory muscle strength. A study reported that low-intensity exercise training improved respiratory function, including %VC, in patients with stable COPD but with reduced MIP (MIP < 60 cm H_2_O) [24]. In addition, it has been reported to significantly improve MIP, respiratory muscle endurance, incremental load pressure, exercise tolerance, Borg scale rating, dyspnea (transition dyspnea index), and health-related QOL in patients with COPD [24]. These data are consistent with the results of this present study in that IMT significantly improved MIP in participants. Considering that dyspnea occurs in patients with advanced lung cancer because of malaise and respiratory muscle fatigue [3], the improvement in MIP may have alleviated participants’ respiratory muscle fatigue and improved their dyspnea.

The duration of IMT loading in this study is also noteworthy. Many previous studies have set a relatively long intervention period (e.g., 2–6 weeks of IMT postoperatively in patients with lung cancer) [19,25,26]. The results were relatively similar to other previous studies, although 4 weeks of IMT had an earlier effect than previous studies suggesting an effect on the respiratory muscles and exercise capacity without adverse events in NSCLC patients who underwent RT [27]. In this study, significant improvement in MIP was observed as early as 1 week after IMT. In addition, a significant improvement in dyspnea was observed between the baseline and week 1. A clear difference between previous studies and this present study is in the use of surgical intervention. Moreover, the manifestation of relatively severe dyspnea and decreased activity, as well as a large number of patients with an emergence period for post-chemotherapy myelosuppression after week 2, could have led to early improvements in MIP and dyspnea. Although most of the patients with lung cancer in this study were treated with radiation and chemotherapy for a period of approximately 2 weeks, the feasibility of this study, which showed efficacy in a relatively short period of time, was considered to be high.

A previous study defined the minimally clinically important difference in rest and exertion mBS scores, which are indicators of dyspnea, as 1 in clinical practice [28]. Current evidence supports IMT as an alternative to aerobic exercise, particularly when time is essential [29]. Therefore, the results of this study suggest that IMT has a high persistence rate and is highly effective for patients with advanced lung cancer and dyspnea who are unable to undergo high-intensity exercise therapy.

### Limitations of the Study

As this was a retrospective cohort study, the sample was small, the results were limited to changes in MIP and dyspnea, and the effects of other indicators were not investigated. In addition, baseline %VC, FVC, and MIP ratings were statistically lower in the IMT + exercise group, which may better reflect the effects of IMT. The MIP after 2 weeks was nearly the same, and more load or more weeks may have been needed to improve MIP in the IMT load + exercise therapy group. The long-term effects of IMT are also unclear. More studies are needed to further investigate and confirm the effects of IMT on QOL and respiratory complications. In addition, dyspnea includes not only measures of inspiratory muscle strength but also the effects of malaise and mental function; however, these latter factors were not assessed in this study. Therefore, studies with rating scales for malaise, dyspnea, and mental functions are desirable.

## 5. Conclusions

This study revealed that IMT load significantly improved MIP and dyspnea at rest and during exertion in patients with advanced lung cancer. Moreover, the effects of IMT load were observed relatively early. These results suggest that IMT is useful and has a high persistence rate in patients who have advanced lung cancer and dyspnea and are unable to undergo high-intensity exercise therapy.

## Figures and Tables

**Table 1:** Clinical characteristics at baseline.

Variable	IMT + Exercise Group (*n* = 31)	Exercise Group (*n* = 35)	*p*-Value
Age (years)	69.1 ± 7.1	70.7 ± 7.4	0.478
Men/women, *n* (%)	16 (52)/15 (48)	21 (60)/14 (40)	0.437
BMI (kg/m^2^)	18.2 ± 1.7	18.3 ± 2.2	0.638
Type of cancer			
Non-small cell lung cancer, *n* (%)	20 (65)	24 (69)	0.732
Small cell lung cancer, *n* (%)	11 (35)	11 (31)	
Cancer stage			
Stage III, *n* (%)	16 (52)	23 (66)	0.177
Stage IV, *n* (%)	15 (48)	12 (34)	
PS	1.2 ± 1.2	1.3 ± 1.0	0.898
BI	92.6 ± 7.7	91.2 ± 6.9	0.515
Medication			
Line	2.7 ± 1.3	2.7 ± 1.1	0.99
Chemotherapy, *n* (%)	9 (29)	15 (43)	0.372
Molecular target drugs, *n* (%)	10 (32)	8 (23)	
ICI, *n* (%)	8 (26)	8 (23)	
Noting, *n* (%)	4 (13)	4 (11)	
Supplemental O_2_, *n* (%)	5 (24)	8 (23)	0.517
Physiologic			
VC (%)	52.6 ± 13.4	56.7 ± 18.7	*p* = 0.04
FVC (L)	1.4 ± 0.3	1.8 ± 0.5	*p* < 0.001
FEV1.0 (%)	68.9 ± 9.1	70.1 ± 14.9	0.746
Outcome			
MIP (cmH_2_O)	33.2 ± 7.2	39.9 ± 11.2	*p* = 0.023
Dyspnea at rest (mBS)	2.3 ± 0.9	2.0 ± 1.0	0.344
Dyspnea on exertion (mBS)	3.8 ± 0.8	3.9 ± 1.0	0.556

Data are counts (percentages) or mean ± SD. Definition of abbreviations: BMI indicates body mass index; PS, Performance status; BI, Barthel index; mBS, modified Borg scale; ICI, Immune checkpoint inhibitor; VC, vital capacity; FVC, forced vital capacity; IMT, Maximal inspiratory pressure.

## Data Availability

Not applicable.
